# Physicochemical and Functional Characterization of Newly Designed Biopolymeric-Based Encapsulates with Probiotic Culture and Charantin

**DOI:** 10.3390/foods10112677

**Published:** 2021-11-03

**Authors:** Awa Fanny Massounga Bora, Xiaodong Li, Lu Liu

**Affiliations:** 1 College of Food Science, Northeast Agricultural University, No. 59 Mucai St., Xiangfang Dist., Harbin 150030, China; fannyatitud@yahoo.fr (A.F.M.B.); liulu89824@163.com (L.L.); 2 Key Laboratory of Dairy Science, Ministry of Education, Northeast Agricultural University, No. 600 Changjiang St., Xiangfang Dist., Harbin 150030, China

**Keywords:** encapsulation, synbiotic, *Lactobacillus acidophilus*, charantin, whey protein isolate, maltodextrin

## Abstract

The identification of novel sources of synbiotic agents with desirable functionality is an emerging concept. In the present study, novel encapsulates containing probiotic *L. acidophilus* LA-05^®^ (LA) and Charantin (CT) were produced by freeze-drying technique using pure Whey Protein Isolate (WPI), pure Maltodextrin (MD), and their combination (WPI + MD) in 1:1 core ratio, respectively. The obtained microparticles, namely WPI + LA + CT, MD + LA + CT, and WPI + MD + LA + CT were tested for their physicochemical properties. Among all formulations, combined carriers (WPI + MD) exhibited the highest encapsulation yields for LA (98%) and CT (75%). Microparticles showed a mean d (4, 3) ranging from 50.393 ± 1.26 to 68.412 ± 3.22 μm. The Scanning Electron Microscopy revealed uniformly amorphous and glass-like structures, with a noticeably reduced porosity when materials were combined. In addition, Fourier Transform Infrared spectroscopy highlighted the formation of strong hydrogen bonds supporting the interactions between the carrier materials (WPI and MD) and CT. In addition, the thermal stability of the combined WPI + MD was superior to that of pure WPI and pure MD, as depicted by the Thermogravimetric and Differential Scanning Calorimetry analysis. More interestingly, co-encapsulation with CT enhanced LA viability (8.91 ± 0.3 log CFU/g) and Cells Surface Hydrophobicity (82%) in vitro, in a prebiotic-like manner. Correspondingly, CT content was heightened when co-encapsulated with LA. Besides, WPI + MD + LA + CT microparticles exhibited higher antioxidant activity (79%), α-amylase inhibitory activity (83%), and lipase inhibitory activity (68%) than single carrier ones. Furthermore, LA viable count (7.95 ± 0.1 log CFU/g) and CT content (78%) were the highest in the blended carrier materials after 30 days of storage at 4 °C. Synbiotic microparticle WPI + MD + LA + CT represents an effective and promising approach for the co-delivery of probiotic culture and bioactive compounds in the digestive tract, with enhanced functionality and storage properties.

## 1. Introduction

The growing awareness of the important role played by the gut microbiome in combating chronic diseases has aroused a great enthusiasm for probiotic organisms. Probiotics are defined as live microorganisms which, when administered in adequate amounts, confer a health benefit to the host [1]. Probiotics such as *L. acidophilus* LA-05^®^ have been attributed a plethora of health benefits which include the enhancement of immunity [2], anti-lipidemic [3], anti-carcinogenic [4], the improvement of gut health [5], etc. Thus, given their potential effects in preventing and possibly treating some chronic diseases, probiotic cultures represent a promising alternative to conventional treatments.

However, despite their numerous health benefits, probiotic organisms are highly sensitive to adverse conditions such as high temperature, acidity, oxidation, etc. [6]. Moreover, it has been reported that probiotic products should contain at least 10^6^ CFU/mL of viable bacteria to claim health properties [7]. Correspondingly, one efficient way to enhance the viability of probiotic organisms is to combine them with prebiotics, thus creating a synergistic system referred to as synbiotic. Hence, the term synbiotic means the association of probiotic cultures with a biological compound (prebiotic) that can selectively enhance its survival to provide health benefits to the host [8]. Diverse mechanisms of action have been proposed to support the synergistic cooperation existing between probiotic and prebiotic. For instance, It was reported that prebiotic might improve probiotic survival by serving as a nutrient, or by producing antimicrobial by-products which reduce the growth of competitive pathogenic bacteria [9].

Charantin, a steroidal saponin from the plant *Momordica charantia*, is one of the lead compounds responsible for the health benefits of the plant, among which are antioxidant, anti-diabetic, anti-inflammatory, anti-cancer, and anti-obesity properties [10,11,12]. Wang et al. [13] reported that a Charantin-rich extract of *Momordica charantia* led to a significant decline in blood glucose, plasma glucose intolerance, and insulin resistance in a mouse model. Additionally, Charantin demonstrated high antimicrobial properties against bacterial species such as gram-positive (Bacillus subtilis), gram-negative (*Pseudomonas aeruginosa*), and fungal strains (Saccharomyces cerevisiae) [14]. Interestingly, recent pieces of evidence suggest that dietary saponins might exhibit a prebiotic effect on probiotic organisms. Hence, Huang et al. [15] reported that ginsenoside saponins promoted the growth of beneficial bacteria such as *Bifidobacterium*, *Lactobacillus*, and *Bacteroides acidifaciens*. Although the prebiotic role of dietary saponins has not yet been established, it was demonstrated that their health-promoting effects might be, in part, through the manipulation of the gut microbiota to the benefit of the host [16]. Therefore, the formulation and characterization of a probiotic-charantin synbiotic in biopolymeric encapsulates is an innovative concept that has not yet been explored.

Microencapsulation is a promising method for the preservation and release of active ingredients in food systems [17]. It presents the advantage of protecting the core material from the external environment, provided that proper encapsulation technique and wall material are chosen. Freeze-drying or lyophilization is preferred to other encapsulation techniques due to its suitability to thermally sensitive compounds. Among the diverse coating materials, Whey Protein Isolate (WPI) was found efficient in enhancing probiotic bacteria survival upon digestion [18], whereas Maltodextrin (MD) has successfully been used to improve cell viability during the freeze-drying process [19]. Finally, it was announced that a sufficient number of dried probiotics could survive storage conditions if an appropriate combination of protein and carbohydrate was selected as carriers [20].

Hence, in the present study, freeze-drying technology was employed for the synbiotic encapsulation of *L. acidophilus* LA-05^®^ and Charantin, using pure WPI, pure MD, and their combination WPI + MD, as wall materials. The Physicochemical parameters of the microparticles including particle size, Zeta potential, moisture content, water activity, and hygroscopicity were determined. In addition, microparticle structure and molecular interaction were examined by Scanning Electron Microscopy (SEM) and Fourier Transform Infrared spectroscopy (FTIR), respectively. Moreover, the thermal stability of the microparticles was assessed via Thermogravimetric and Differential Scanning Calorimetry analysis (TGA-DSC). Finally, the microparticles in-vitro functionality and storage stability were also carried out.

## 2. Material and Methods

### 2.1. Material

Commercial probiotic culture of *L. acidophilus* LA-05^®^ was obtained from Chr. Hansen (Hoersholm, Denmark) and Charantin (95% *w*/*w*) was acquired from Yueyuan natural Co., Ltd. (Xi’an, China). Whey protein isolate (92%) and maltodextrin were purchased from Beijing Milky Way Trade Corp., Ltd. (Beijing, China) and Baolingbao Biology Co., Ltd. (Shandong, China), respectively. Man, Rogosa, and Sharpe (MRS) broth, and MRS agar were acquired from Hangzhou Baisi Biotechnology Co., Ltd. (Hangzhou, China). The 2,2′-diphenyl-1-picrylhydrazyl (DPPH) and α-amylase reagents were provided by Biotopped Life Sciences Co., Ltd. (Beijing, China).

### 2.2. Bacteria Preparation

Bacteria were prepared following the method of Bora et al. [21]. Briefly, a freeze-dried culture of *L. acidophilus* LA-05^®^ was inoculated into Man, Rogosa, and Sharp (MRS) broth and incubated at 37 °C for 24 h. Bacterial cells were harvested by centrifugation (Allegra™64R, Beckman, Germany) at 3000× *g*, 4 °C for 10 min, and washed twice with 0.9% sterile saline water.

### 2.3. Wall Materials Preparation

The different wall materials (WPI, MD and WPI + MD) were prepared based on the method of Bora et al. [22]. Briefly, pure WPI and pure MD were individually dispersed in distilled water and dissolved at 40 to 60 °C with constant magnetic stirring for 30 min. To obtain the blended carrier material, equal quantities of WPI and MD were simultaneously homogenized with an overhead stirrer (Wiggens model WB2000-D) operating at 100 rpm for 5 min.

### 2.4. Microparticles Formulation

The bacterial count was performed based on the Colony Forming Unit method. Hence, the prepared bacterial suspension obtained in Section 2.2 was serially diluted (1/100), and 100 µL of each dilution was spread onto agar plates. After overnight incubation at 37 °C, colonies were counted on the plates to obtain a final concentration of at least 10 log CFU/mL, corresponding to the number of bacterial colonies counted on plate × dilution factor/Volume of culture plate. Then, bacterial cells accounting for about 10 log CFU/mL and Charantin (5% *w*/*w*) were simultaneously added to the different prepared wall material (WPI, MD and WPI-MD) in a core-to-wall ratio of 1:1 (20% *w*/*w*), respectively. The different preparations were homogenized and kept at −4 °C for 24 h. The freeze-drying process was carried out using a pilot-scale freeze-dryer (Christ D-37520, Osterode, Germany) at 4 °C for 24 h. The obtained freeze-dried microparticles were aseptically kept in aluminum foil and stored at 4 °C until further characterization.

### 2.5. Encapsulation Efficiency (EE)

#### 2.5.1. Bacteria Encapsulation Efficiency

Encapsulated bacteria were released by suspending 500 mg of microparticles in 5 mL of 2% sterile sodium citrate solution adjusted to pH 6.0, followed by vigorous stirring for 10 min [23]. The microorganisms were dispersed on MRS agar plates and incubated for 48 h at 37 °C in anaerobic jars containing anaerogen sachets (Oxoid). The bacterial count was determined by the Colony-Forming Unit method as described in Section 2.4, and the encapsulation efficiency was calculated as follow:

EE (*L. acidophilus*) = (N/N_0_) × 100 (1)

where N is the number of viable cells released from the microparticles and N_0_ the number of cells before microencapsulation.

#### 2.5.2. Charantin Encapsulation Efficiency

Charantin encapsulation efficiency was determined according to an adapted method of Nirupama et al. [24]. Briefly, 0.5 mg of freshly prepared microparticles was suspended in 5 mL of sodium citrate solutions (2%, pH 6.0) and stirred for 10 min. The samples were centrifuged (6121× *g*/4 °C/3 min) to remove bacterial cells from the solutions. Charantin content in the supernatant was observed via UV-Vis spectrophotometry at wavelength 281 nm. A solution of Phosphate buffer (0.1 M, pH 6.8) was used as blank and a prepared solution of 10 µg/mL of Charantin (pH 6.8) served as a buffer solution. Charantin encapsulation efficiency was calculated as follow:

EE (Charantin) = (C/C_0_) × 100 (2)

where C is the content of Charantin in microparticles and C_0_ the content of Charantin before microencapsulation.

### 2.6. Fourier Transform Infrared Spectrometer (FTIR)

The chemical interaction between wall and core materials was investigated via FTIR spectroscopy (Shimadzu, Japan), operating at a resolution of 2 cm^−1^ over the wavenumber area of 4000–400 cm^−1^.

### 2.7. Particle Size Measurement and Zeta Potential

Particle size measurement was made using the Mastersizer 2000 laser diffraction equipment (Malvern, Germany). The carrier fluid used for the microparticles was Isobutanol and the refractive index was 1.394. The particle size measurements were reported as volume-weighted mean diameter d (4, 3).

### 2.8. Scanning Electron Microscopy (SEM)

The morphological characterization of the microparticles was made by scanning electron microscopy (Hitachi S-4300/N Field Emission, Variable Pressure, Japan) operating at an accelerating voltage of 10.0 kV. The samples were placed on a double-sided adhesive carbon tab and coated with gold.

### 2.9. Physicochemical Characteristics of the Synbiotic Microparticles

The moisture content analysis was performed following the AOAC method [25], and the water activity was determined using an AquaLab Vapor Sorption Analyzer (VSA, Meter Group, Pullman, WA, USA) at 25 °C.

The hygroscopicity was determined according to Cai and Corke [26]. The samples (1 g) were transferred into a desiccator containing a saturated solution of sodium chloride (75.3%) at 25 °C. After 1 week, the samples were weighed and their hygroscopicity was expressed in percentage (%) of adsorbed moisture.

### 2.10. Thermogravimetric and Differential Scanning Calorimetry Analysis TGA-DSC

A TGA/DSC thermogravimetric analyzer (STA449F3, Netzsch, Germany) was used to investigate the thermal stability of the samples. Samples (3 g) were sealed in aluminum pans, and a sealed empty pan was used as a reference. The heating rate and heating temperature range were 10 °C/min and 30–500 °C, respectively.

### 2.11. In Vitro Stability and Cells Surface Hydrophobicity

#### 2.11.1. In Vitro Stability of the Encapsulated Probiotics

The in vitro stability of the samples was carried out according to the method proposed by Minekus et al. [27]. The samples were sequentially subjected to simulated salivary fluid (SSF), simulated gastric fluid (SGF), and simulated intestinal fluid (SIF). Details of the solutions are given in Table 1. Samples were mixed with the SSF (1:1, (*wt*/*wt*)) and the solution was readjusted to pH 7 using 1.0 M NaOH solution. The obtained slurry was let to incubate at 37 °C for 2 min under constant agitation. Then, the SGF (1:1 (*v*/*v*)) and a solution of porcine pepsin (3.8 mg/mL) were added to the oral bolus. The mixture was adjusted to pH 3 using a solution of hydrogen chloride (1.0 M) and incubated at 37 °C for 2 h with constant agitation. Thereafter, the obtained gastric chyme was mixed with SIF (1:1 (*v*/*v*)), porcine bile extract (37.8 mg/mL), and porcine pancreatin (16.25 mg/mL). The resulting intestinal chyme was incubated (37 °C, pH 7) for 2 h. At the end of each stage, aliquots of each sample (1 mL) were diluted in 9 mL of sterile peptone water (0.2%, *w*/*v*) and placed in a stomacher for 30 s. The bacterial count was obtained by serially diluting the suspensions, followed by incubation in MRS agar plates at 37 °C for 48 h. CT content was determined via UV-Vis spectrophotometry at wavelength 281 nm following the method described in Section 2.5.2.

#### 2.11.2. Cell Surface Hydrophobicity (CSH)

The surface hydrophobicity of the free and encapsulated bacteria cells was determined using the microbial adhesion to hydrocarbons method according to Petrova et al. [28], with some modifications. The microparticles were mixed with a sodium phosphate buffer (0.4 M) solution and stirred to allow the release of bacteria cells. Then, the bacterial suspension was centrifuged (14,000× *g* for 5 min at 4 °C), washed twice with 50 mM K_2_HPO_4_ (pH 7.0), and resuspended in 50 mM K_2_HPO_4_ to realize an absorbance value of 0.5 at 560 nm. 5 mL of the suspension was mixed with 1 mL of *n*-hexadecane (1:6). The mixture was vortexed for 2 min and incubated at room temperature for 1 h. After complete separation of the two phases, CSH was determined by UV–Vis spectrophotometer as follows:

CSH (%) = [(A_0_ − A)/A_0_] × 100 (3)

where A_0_ and A are absorbances before and after extraction with *n*-hexadecane, respectively.

### 2.12. Antioxidant Activity

The antioxidant activity of the microparticles was evaluated using the 2,2-diphenyl1-picrylhydrazyl free radical scavenging assay (DPPH). Briefly, 200 mg of samples were mixed with 5 mL of 0.1 mM DPPH methanolic solution. The reaction mixtures were incubated in the dark at ambient temperature for 30 min, and their absorbance was measured at 517 nm. The percentage of DPPH free radical scavenging activity of microparticles was determined using the following equation:

DPPH scavenging activity (%) = (A_DPPH_ − A_S_/A_DPPH_) × 100 (4)

where A_DPPH_ is the absorbance value of the DPPH radical methanolic solution and A_S_ is the absorbance value of the sample.

### 2.13. α-Amylase and Lipase Inhibitory Activities

The α-amylase inhibitory assay [29] was used to investigate the potential anti-diabetes activity of the microparticles. A volume of 100 μL of powder solutions (10 mg/mL in 0.1 M PBS, pH 6.9) was added to 100 μL of 13-U/mL α-amylase solution (1 mg/mL in 0.1 MPBS, pH 6.9). The mixture was allowed to react for 10 min at 37 °C, and 100 μL of 1% soluble starch solution in PBS, previously boiled for 5 min, was added to each tube and incubated for another 10 min. Finally, a volume of 200 μL of dinitrosalicylic acid reagent was added to each test tube, followed by heating at 100 °C for 5 min in a water bath. Further, the samples were diluted with 2 mL of distilled water, and absorbance was read at a wavelength of 540 nm. Enzyme inhibition was calculated according to the following equation:

α-amylase inhibitory activity (%) = (OD_c_ − OD_s_/OD_control_) × 100% (5)

where OD_C_ is the absorbance value of the control and OD_S_ is the absorbance value of the sample.

The potential anti-obesity activity of the microparticles was investigated based on the lipase inhibitory activity according to Costamagna et al. [30]. Briefly, The enzymatic hydrolysis of *p*-nitrophenyl palmitate to *p*-nitrophenol was calculated by mixing the microparticle (10 mg/mL) with a concentration of Lipase (1.0 mg/mL). The mixture was kept at 25 °C for 5 min, then 330 µL of the substrate was supplemented with Triton X-100 (0.6% *w*/*v*) and 0.15% Arabic gum was added to the mixture. The solution was incubated at 37 °C for 20 min and absorbance was read at a wavelength of 400 nm. Enzyme inhibition was calculated according to the following equation:

lipase inhibitory activity (%) = (OD_c_ − OD_s_/OD_control_) × 100% (6)

where OD_C_ is the absorbance value of the control and OD_S_ is the absorbance value of the sample.

### 2.14. Microparticles Storage Stability

The stability of the probiotic cultures and the content of Charantin were evaluated under different storage conditions (4 °C and 25 °C) during 30 days, in sterile glass vials. Samples (1 g) were collected every week for assessing the viability of free and encapsulated bacteria by pour plate counting following the method described in Section 2.4., and the content of Charantin via UV-VIS spectrophotometry according to the methods detailed in Section 2.5.2.

### 2.15. Statistical Analyses

Tests were performed in triplicate, and data were expressed as mean ± standard deviation. The statistical analysis was performed by one-way ANOVA using GraphPad Prism software (San Diego, CA, USA), and the differences at *p* < 0.05 were considered significant.

## 3. Results and Discussion

### 3.1. Encapsulation Efficiency

**Figure 1:**
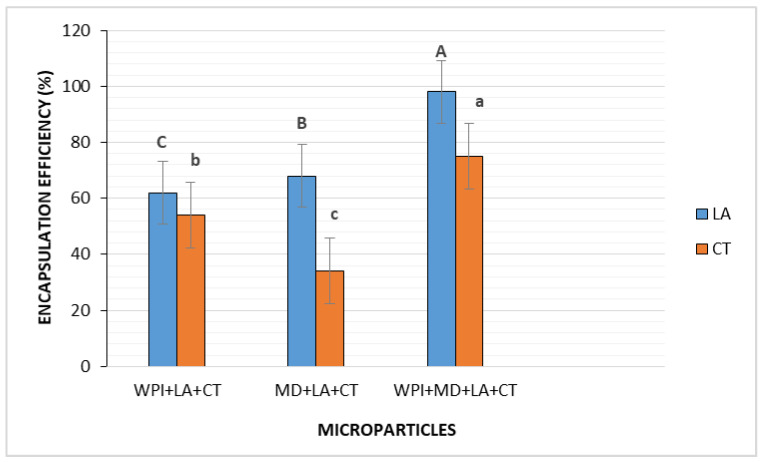
Encapsulation efficiency of microparticles. WPI + LA + CT, MD + LA + CT, and WPI + MD + LA + CT. WPI + LA + CT: Whey protein isolate + *L. acidophilus* + Charantin; MD + LA + CT: Maltodextrin + *L. acidophilus* + Charantin; WPI + MD + LA + CT: Whey protein isolate + Maltodextrin + *L. acidophilus* + Charantin. Different letters above each column represent significant differences in means (*p* < 0.5).

The results of the encapsulation efficiency reported in Figure 1 reveal that the carrier materials had a significant impact on the encapsulation efficiency. Hence, among all formulations, the formulation of the combined materials (WPI + MD) exerted the highest encapsulation yields for both LA (98%) and CT (75%), compared to single-carrier encapsulates WPI (62%, 54%) and MD (68%, 34%), respectively. The obtained results were in agreement with previous studies reporting higher encapsulation yield when a blend of biopolymers was used for the co-encapsulation of probiotic Lactobacillus acidophilus and blackberry juice [31]. Similarly, a protein-polysaccharides complex coacervate constituted of whey protein isolate and gum arabic was found significantly superior to single wall material, in encapsulating probiotic bacteria and omega-3 fatty acids [32]. Indeed, protein–polysaccharides complexes induced by the addition of small amounts of salt, or in our case increase of temperature, contribute to the formation of stronger and more rigid structures than single polymers [33]. This stronger structure may have been favored by hydrophobic interactions occurring in the protein-polysaccharides complex. In this regard, Gulão et al. [34] observed the formation of aggregates between lactoferrin and gum arabic at 80 °C, suggesting the participation of hydrophobic interactions by exposing the hydrophobic groups of the protein after a conformational change promoted by the increase in temperature. On the other hand, the hydrophilic nature of MD may have acted as a stabilizing agent by remaining in the aqueous phase during complexation, thus helping in controlling the aqueous phase rheology such as thickening and gelling [35]. The aforementioned results attest to the suitability of the blend of biopolymers WPI + MD for the encapsulation of probiotic-bioactive compounds synbiotic systems.

### 3.2. Fourier Transform Infrared Spectrometer (FTIR)

**Figure 2:**
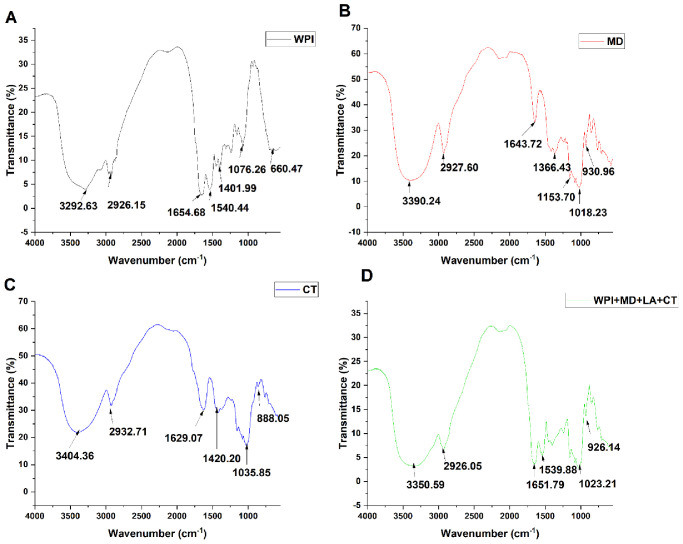
FTIR spectra of WPI (**A**), MD (**B**), CT (**C**), and WPI + MD + LA + CT (**D**).

FTIR analysis was carried out in order to investigate the interactions between the different carriers and the core materials (Figure 2). The FTIR spectra of WPI (Figure 2A) exhibited a typical peak at 1540.44 cm^−1^ which corresponds to the amid I regions composed of C=O stretching. The peak at 1654.68 cm^−1^ was representative of the amide II region, which is composed of C–N stretching, coupled with N–H bending [36]. The peaks around 1076.26 and 1401.99 cm^−1^ regions revealed N–H groups related to the amide I region. The peak expressed at 2926.15cm^−1^ was ascribed to C–H stretching band and O–H, and the peak at 3292.63 cm^−1^ was attributed to N–H stretching (amide A). Regarding MD (Figure 2B), the peaks in the range of 1018.23–1153.70 cm^−1^ were attributed to the carbonyl group [37]. The peaks observed at 1643.72 cm^−1^ and 1366.43 cm^−1^ were imputed to the asymmetric and symmetric stretching vibration of the carboxylic acid salt –COO, and the peaks ranging from 2927.60 to 3390.24 cm^−1^ were an indication of stretching vibrations of CH_2_ and CH_3_ groups. The FTIR spectra of free Charantin (Figure 2C) presented a typical peak at 1035.85 cm^−1^ which was related to stretching C–O, and a peak at 1629 cm^−1^ which corresponded to C=C groups [38]. Besides, the peak at 2932.71 cm^−1^ was attributed to asymmetric stretching vibrations of the CH_2_ groups of the alkyl chain [39], and the peak observed at 3404 cm^−1^ was associated with hydroxyl groups. In comparison to the FTIR spectrum of WPI, MD, and CT, the FTIR spectra of WPI + MD + LA + CT (Figure 2D) presented considerable structural changes. Hence, it can be seen that the peak at 1654.68 cm^−1^ (amid I) in the WPI spectra, shifted to lower wavenumber (1651.79 cm^−1^) in the WPI + MD + LA + CT, spectra, while the peak at 1540.44 cm^−1^ (amid II) decreased to 1539.88 cm^−1^ in the WPI + MD + LA + CT spectra. Those shifts were attributed to glycation taking place during the heating stage of the conjugate formation [40]. It was concluded that the conjugation process induced a change in the protein secondary structure in WPI + MD + LA + CT. Similarly, peaks at 3390.24 cm^−1^ and 2927.60 cm^−1^ of the MD spectra shifted to 3350.59 cm^−1^ and 2926.05 cm^−1^ in the WPI + MD + LA + CT spectra, respectively. More, MD stretching vibration band ─COO^−^ disappeared from the spectra of blended carrier WPI + MD + LA + CT, suggesting its electrostatic interaction with the amine groups of WPI (─NH3^+^), which was in support of the zeta potential analysis [41]. Furthermore, those observed changes were an indication that the interactions between the carrier materials (WPI and MD) and CT occurred through hydrogen bonds. Finally, the absence in the peaks 2932.71 cm^−1^ and 1629.07 cm^−1^ of the free CT from the WPI + MD + LA + CT spectra indicated a successful encapsulation of CT into the blended materials.

### 3.3. Particle Size and Zeta-Potential

The particle size is an important factor that can affect the desirability of a food product, as small particle sizes produce finer powders that are more palatable. The particle size of the different microparticles is reported in Table 2. The mean particle diameter d (3, 4) of all the produced microparticles ranged from 50.393 ± 1.26 to 68.412 ± 3.22 μm. In comparison to other methods on the co-encapsulation of probiotics and bioactive compounds, our obtained microparticles were relatively smaller than the average range of microparticles reported by previous studies. For example, Shinde et al. [42] obtained diameters ranging from 423 to 486 μm for the co-extrusion encapsulation of probiotic *Lactobacillus acidophilus* with apple skin polyphenols. In addition, microparticles ranging from 127.5 to 234.6 μm were produced for the co-encapsulation of *Lactobacillus acidophilus* and different prebiotic agents by external ionic gelation followed by freeze-drying [43]. In addition, Gaudreau et al. [44] reported microparticles ranging from 81 to 130 µm when probiotic *Lactobacillus helveticus* was co-encapsulated with green tea extract in calcium pectinate microparticles by the emulsification/internal gelation technique. More recently, the microencapsulation of probiotic *Lactobacillus paracasei, L. casei* 431, and anthocyanins from black rice (*Oryza sativa* L.) in a biopolymer matrix composed of milk proteins and inulin by freeze-drying resulted in microspheres ranging from 128.25 to 263.25 µm for the ethanolic extract and 339.95 to 346.21 µm for the aqueous extract [45]. Therefore, our obtained microparticles would be desirable as a food additive as studies have revealed that microparticles presenting a diameter less than 100 µm would not have an important effect on the texture of the food [46].

The Zeta-potential of the different microparticles was determined to examine their electrostatic nature and further investigate the protein-polysaccharides complex WPI-MD with regard to electrostatic interaction (Table 1). Microparticles Zeta-potential values are reported in Table 2. Microsphere MD exerted a negative charge (−2.31 ± 0.3), while microspheres WPI and WPI-MD both exerted positive charges (2.65 ± 0.5) and (4.92 ± 0.2), respectively. The negative charge exerted by MD is typical of polysaccharides which tend to become anionic at a pH range higher than their pKa [47]. The positive charge observed for WPI and the blended formulation WPI-MD can be attributed to the protonic nature of proteins, which in the latter case dominated the electrostatic nature of the blended microparticles. Similar results were obtained for microparticles formulated with a blend of MD and WPC (Whey Protein Concentrate) containing probiotic *Lactobacillus acidophilus* and Blackberry juice [31].

### 3.4. Particle Morphology

**Figure 3:**
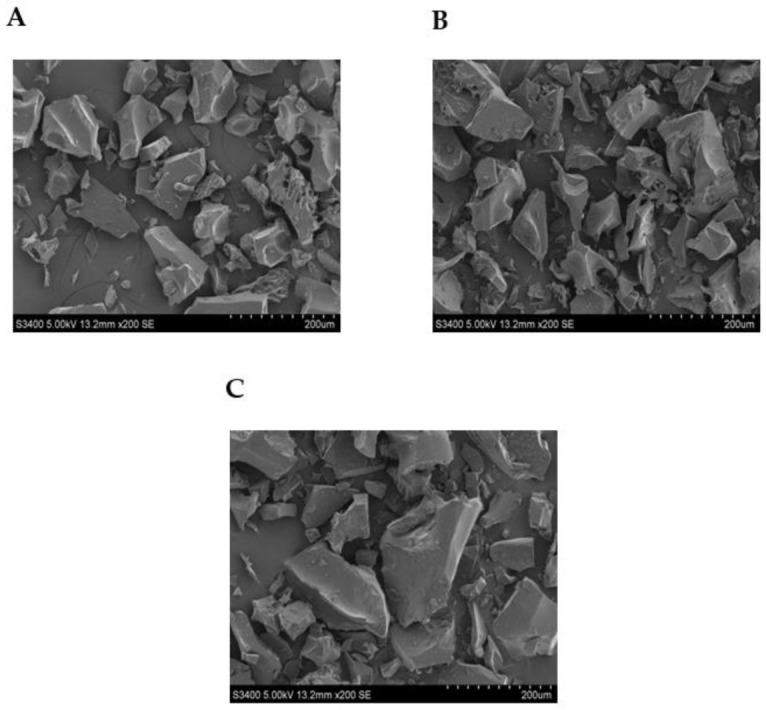
SEM images of freeze-dried microparticles. WPI + LA + CT (**A**), MD + LA + CT (**B**), WPI + MD + LA + CT (**C**). WPI + LA + CT: Whey protein isolate + *L. acidophilus* + Charantin; MD + LA + CT: Maltodextrin + *L. acidophilus* + Charantin; WPI + MD + LA + CT: Whey protein isolate + *L. acidophilus* + Maltodextrin + *L. acidophilus* + Charantin.

SEM images of freeze-dried microcapsules prepared with different wall material formulations are shown in Figure 3. Microparticles appeared fragmented, irregular in shape, with a flask-like structure. Similar structures for freeze-dried particles have been reported by previous studies [48,49,50]. The unevenness of the microparticles shape was attributed to the non-uniform drying of the various parts of the liquid droplets during the early stages of the freeze-drying process [51]. Moreover, the observed porosity was linked to the removal of water by sublimation occurring during the freeze-drying process, which often results in the creation of highly porous structures [52]. Besides, it is to be noted that different carrier materials affected the surface morphology of the microparticles. MD + LA + CT microparticles (Figure 3B) presented more fragmented structures, which might have been caused by the syneresis of polysaccharides microgel during freeze-drying. Compared to other encapsulates, WPI + MD + LA + CT (Figure 3C) presented a smoother and less porous surface. It was concluded that a more uniform structure was obtained for microparticles WPI + MD + LA + CT as a result of the strong binding properties of the two biopolymers as revealed by the FTIR analysis. In addition, the conjugation of WPI-MD may have exhibited higher gelling properties, thus reducing the porosity upon drying. In this regard, it was reported that the gel-forming properties of proteins can be enhanced by conjugation with maltodextrin [53]. Finally, the electrostatic affinity from the opposite charges interactions between the two biopolymers WPI and MD may have also contributed to a more dense and uniform stru.

### 3.5. Physicochemical Characteristics

The results of the physicochemical analysis carried out on the samples are reported in Table 2. The parameters tested were the moisture content, water activity, and hygroscopicity. Moisture content, which is defined as the portion (weight) of water contained in a product, is a significant factor that affects the long-term stability and shelf life of powdered products. Hence, it is necessary to accurately determine and control its value in manufacturing powdered products. The moisture content of the obtained microparticles varied from 3.25 to 4.05%. In general, powdered foods with moisture content <4% are considered suitable [54]. Therefore, except for microparticles WPI-LA-CT which had a moisture content of 4.05 ± 0.3, our microparticles presented desirable moisture content values. The highest moisture content obtained for microparticles WPI-LA-CT can be related to protein high water retention capability in the amorphous state [55]. On the other hand, the lowest moisture content observed for microparticles MD-LA-CT was attributed to MD non-hygroscopic nature.

Water activity is an important parameter that indicates the amount of “free water” available in a product. It is critical to maintain the water activity of the food products within the acceptable range to ensure their safety and prevent undesirable microbial growth. As seen in Table 1, the water activity of the microparticles ranged from 0.22 ± 0.1 to 0.59 ± 0.1. The values of water activity of the obtained microparticles were within the pre-established range of optimal water activity for the stability of freeze-dried probiotics 0.07–0.2 [56].

The microparticles were also tested for their hygroscopicity, which is the tendency of powdered foods to attract or hold water from ambient moisture. Hygroscopicity is a key factor that affects the shelf-life stability and the portability of powdered foods, as powders with high hygroscopicity tend to agglomerate. The hygroscopicity of the microparticles ranged from 7.39 to 12.31%. Previous studies reported different values for hygroscopicity ranging from 11.92% to 14.25% for freeze-dried samples encapsulated in biopolymeric matrices [57]. Based on the results obtained from the different physicochemical tests carried out on the samples, it can be concluded that the microparticles meet the industry quality standards as potential powdered food products.

### 3.6. Microparticles Thermal Stability

**Figure 4:**
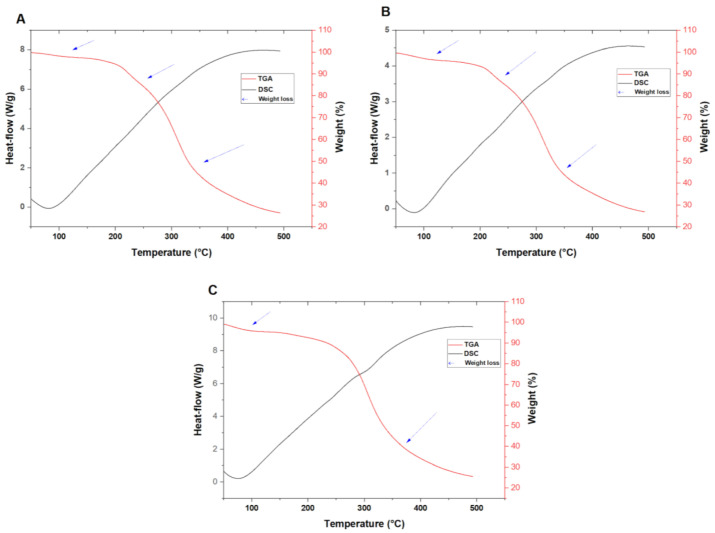
TGA-DSC curves of microparticles WPI + LA + CT (**A**), MD + LA + CT (**B**), WPI + MD + LA + CT (**C**). WPI + LA + CT: Whey protein isolate + *L. acidophilus +* Charantin; MD + LA + CT: Maltodextrin + *L. acidophilus* + Charantin; WPI + MD + LA + CT: Whey protein isolate + Maltodextrin + *L. acidophilus* + Charantin.

Food products undergo diverse processes such as freezing, heating and drying to be transformed into finished goods with desirable qualities. Processes such as heating affect the physicochemical characteristics of food products. TGA and DSC analysis allow the measurement of the weight-loss changes and heat flow in a product, as a function of temperature or time, in a controlled atmosphere. The results of the TGA-DSC analysis of the different samples are depicted in Figure 4. Wall material formulation had a significant impact on the weight-loss behavior of the microparticles. The TGA curves of the single-walled microparticles both presented three main weight loss regions. WPI + LA + CT (Figure 4A) main weight loss regions are from 35 to 185 °C, from 200 to 335 °C, and from 360 to 500 °C. MD + LA + CT (Figure 4B) main weight loss regions are from 32 to 205 °C, from 230 to 268 °C, and from 340 to 500 °C. Comparatively, WPI + MD + LA + CT (Figure 4C) presented two main weight-loss regions from 55 to 230 °C and from 260 to 500 °C. For all microparticles, the weight loss occurring from 30 to approximately 200 °C was attributed to surface water loss, and the weight loss observed from 200 to 500 °C was associated with the decomposition of materials. It is to be noted that the TGA curve of WPI + MD + LA + CT presented a single-stage decomposition compared to WPI + LA + CT and MD + LA + CT curves which all revealed multistage decomposition. Those observations indicate that WPI + MD + LA + CT microparticles demonstrated higher thermal stability compared to WPI + LA + CT and MD + LA + CT microparticles. Moreover, the DSC curves of all microparticles revealed a broad endothermic peak ranging from 30 to approximately 130 °C which was assigned to evaporation of absorbed water [58].

### 3.7. Microparticles Functional Properties

**Figure 5:**
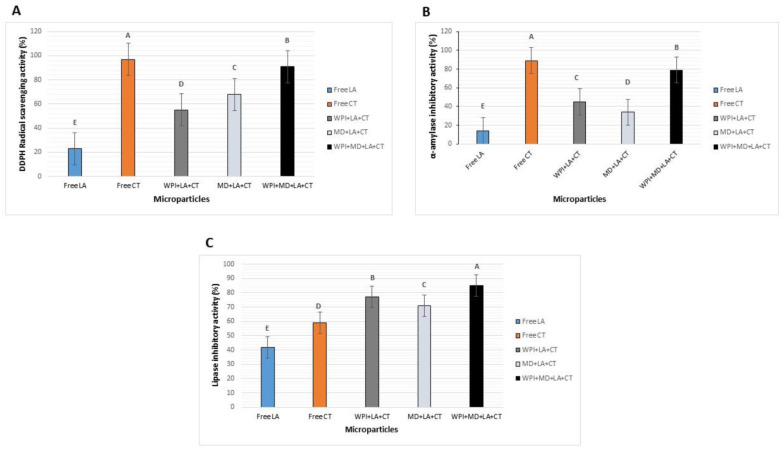
Microparticle functional properties: antioxidant activity (**A**), antidiabetic activity (**B**), and antiobesity activity (**C**). WPI + LA + CT: Whey protein isolate + *L. acidophilus +* Charantin; MD + LA + CT: Maltodextrin + *L. acidophilus +* Charantin; WPI + MD + LA + CT: Whey protein isolate + *L. acidophilus* + Maltodextrin + *L. acidophilus +* Charantin. Data represent means ± standard deviation. Different letters above each column represent significant differences in means (*p* < 0.5).

Oxidative stress causes cell damage due to the presence of free radicals. It is recognized to play a causal role in the pathogenesis of many chronic diseases. Therefore, the consumption of compounds presenting antioxidant properties may lower the risk of health disorders. The different microparticles were assessed for their antioxidant activities and the results are shown in Figure 5A. The two controls, namely free LA and free CT, exhibited the lowest (23%) and the highest (97%) antioxidant activities, respectively. Indeed, *Lactobacilli* strains have been accredited with antioxidative activities [59]. Cecchi et al. [60] study revealed that *Lactobacilli* strains had higher total antioxidant activity than other examined strains. In addition, Lin et al. [61] found out that Lactobacillus acidophilus possesses radical scavenging ability, which contributes to the antioxidative effect. The highest antioxidant activity of free CT was anticipated as previous studies have demonstrated the high antioxidant capacity of CT [62]. It is to be noted that carrier materials had a significant impact on the antioxidant activity of microparticles. Hence, among the encapsulated samples, WPI-MD-LA-CT presented the highest antioxidant activity (91%), followed by MD-LA-CT (68%), and WPI-LA-CT (55%). The higher antioxidant activity exhibited by the blended carrier encapsulates was attributed to the combined antioxidant activities of the different core materials. Moreover, this finding also indicates that blended materials provided superior protection to LA and CT, which in return exhibited a higher scavenging activity.

Diabetes mellitus, commonly known as diabetes, is a metabolic disease that causes high blood sugar. The cost associated with conventional treatments and long-term complications represents an enormous economic burden for individuals and public health systems worldwide. Therefore, the development of cost-effective food products presenting curative and/or preventive properties against diabetes is needed. The samples were tested for their potential anti-diabetes properties and the results were expressed in Figure 5B. Free CT exhibited the highest anti-diabetes activity (89%) as it was previously demonstrated that CT possesses putative anti-diabetes activity [63]. On the other hand, free LA presented the lowest anti-diabetes activity (14%). Among the encapsulated samples, blended microparticles WPI-MD-LA-CT exhibited the highest anti-diabetes activity (79%) compared to MD-LA-CT (34%), and WPI-LA-CT (45%), demonstrating the higher protective capability of the blend of materials to maintain the core agents bioactivities. Moreover, the addition of CT enhanced the probiotic antioxidant activity.

Obesity is defined as an abnormal or excessive fat accumulation that is generally caused by inappropriate eating habits and an unhealthy lifestyle. One prolific way of fighting obesity would be to decrease the absorption of fats through the action of lipase inhibitors [64]. Hence, the formulation of food products should be oriented toward the incorporation of compounds presenting lipase inhibitory activity. The results of the lipase inhibitory activity assay are shown in Figure 5C. Free cells demonstrated a lipase activity of (42%), which was in agreement with previous studies reporting that lactic acid bacteria, among which is *L. acidophilus,* demonstrated pancreatic lipase inhibitory activities in vivo [65]. Reamtong et al. [66] found that probiotic *L. acidophilus* improved the lipid profile in high-fat diet-fed rats. Their study revealed that lipase expression was downgraded in high-fat diet rats fed with probiotic *L. acidophilus*. Free CT had lipase inhibitory activity of 59%. This result can be correlated to the fact that saponins from *M. charantia* were found to inhibit pancreatic lipase activity [67]. Comparatively, microparticles samples exhibited higher lipase inhibitory activity than free LA and free CT. In this regard, some studies have revealed that the pancreatic lipase inhibitory activity of saponins was heightened by gut stimulation [68]. In addition, WPI-MD-LA-CT had the highest lipase inhibitory activity (85%), followed by WPI-LA-CT (77%), and MD-LA-CT (71%). It was hypothesized that carrier materials contributed to this effect by simultaneously exhibiting anti-obesity activities. Furthermore, Yoda et al. [69] reported that a combination of probiotics and whey proteins enhanced the anti-obesity effects of calcium and dairy products during nutritional energy restriction in aP2-agouti. Moreover, Boscaini [70] observed the effect of WPI on body weight, adipose tissue, intestinal-related functions, and gut microbiota of C57BL/6 mice fed with a high-fat diet (HFD). The results of this study showed that WPI decreased body weight and adiposity, which might be caused by an increase in adipose tissue catabolism. In light of the obtained results, the synbiotic system WPI-MD-LA-CT presents desirable functionality. Nonetheless, further investigation implicating in vivo and clinical studies is needed to predict its preventive or curative effect on chronic diseases.

### 3.8. Particles In Vitro Stability and Cell Surface Hydrophobicity (CSH)

**Figure 6:**
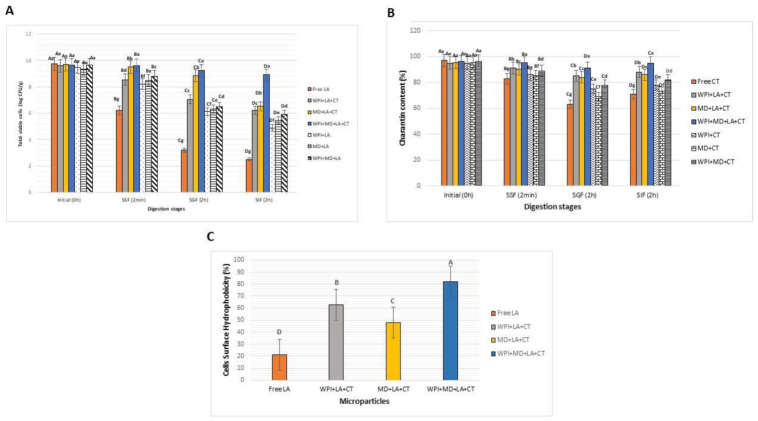
In vitro stability of LA (**A**), CT (**B**) content, and cells surface hydrophobicity of LA (**C**). WPI + LA + CT: Whey protein isolate + *L. acidophilus* + Charantin*;* MD + LA + CT: Maltodextrin + *L. acidophilus* + Charantin; WPI + MD + LA + CT: Whey protein isolate + *L. acidophilus* + Maltodextrin + *L. acidophilus* + Charantin. Different uppercase letters above each column represent significant differences of samples between stages (*p* < 0.5), and lowercase letters above each column indicate significant differences between samples within a stage (*p* < 0.5).

Microparticles were subjected to simulated sequential digestion (SSF, SGF and SIF) to assess the effect of encapsulation on LA viability and CT content upon digestion (Figure 6). At the oral stage (Figure 6A), encapsulated bacteria presented higher survival than free cells, and CT content (Figure 6B) was higher in encapsulated samples than in free samples. At the gastric stage (Figure 6A), free cells had a poor survival as compared to encapsulated cells, and CT content (Figure 6B) drastically dropped in free samples compared to encapsulated samples. Moreover, blended carrier materials WPI + MD provided the highest protection for both LA and CT. At the intestinal stage (Figure 6A), free cells showed no survival as opposed to encapsulated cells which were higher in WPI + MD (8.91 ± 0.3 log CFU/g), followed by MD (6.13 ± 0.4 log CFU/g), and WPI (5.45 ± 0.3 log CFU/g). The obtained results were in agreement with previous studies that found out that protein-polysaccharides conjugates provided higher protection to probiotic cells than single biopolymers wall materials [71]. This superior performance of the blended carrier materials could be attributed to its higher gelling properties which reduced the digestive enzyme penetration. Besides, the binding of carrier materials through strong hydrophobic interactions may have limited pepsin degradation which is usually dependent on the cleavage of the peptide bonds via hydrophobic amino acids interactions [72]. Moreover, probiotics encapsulated with CT demonstrated higher survival than singly-encapsulated ones, suggesting a possible prebiotic effect of CT on LA. In this respect, some studies suggest a possible prebiotic activity of saponins on probiotic organisms. For example, it has been reported that ginsenoside saponins promoted the growth of beneficial bacteria such as *Bifidobacterium*, *Lactobacillus*, *Bacteroides acidifaciens* [15]. Finally, CT content in the encapsulated samples showed an increase at the end of the intestinal stage. The high retention of CT (95%) in co-encapsulated samples may have been promoted by the synergistic effect of probiotic bacteria which were more efficient in this case than free cells. Indeed, it has been revealed that the gut microbiome can induce a series of structural changes in saponins, which generate secondary products presenting a better bioavailability than the original saponins [73]. Blended carrier material allowed higher retention of LA and CT content throughout the digestive tract which is an indication of its suitability for effective co-delivery of probiotic and bioactive compounds.

The CSH of probiotic bacteria is an indication of their adhesion capability to the host epithelium cells to colonize the gastrointestinal tract [74]. Considering that *Lactobacilli* strains adhesion capability can be altered through exposure to various environmental stresses including cold, heat, acid, and starvation [75], it is important to determine the protective capacity of carrier materials on the adhesion properties of the cells as affected by the encapsulation process. In this study, the CSH was determined based on the microbial adhesion to hydrocarbons (MATH) method which is one of the most common methods used to determine the hydrophobicity of cell surfaces [76]. Compared to other methods, the adhesion to hydrocarbons (MATH) presents the advantage of being easy to handle with a low operational cost [77]. The MATH assay is based on determining the adhesion of microorganisms to organic solvents (e.g., *n*-hexadecane, *p*-xylene, *n*-octane) after a short mixing with aqueous cell suspensions and subsequent phase separation. The results of the cell surface hydrophobicity are depicted in Figure 6C. It can be seen that free cells exerted a poor CSH (21%) compared to encapsulated microparticles. Oppositely, encapsulation enhanced the CSH of probiotics as proved by the higher CSH exerted by WPI-MD-LA-CT (82%), WPI-LA-CT (63%), and MD-LA-CT (48%). Besides, the high CSH of WPI-MD-LA-CT can be explained by the fact that blended formulations provided stronger protection to the probiotics cells in the in vitro digestive model, which in return enabled a higher adhesion capability marked by a high CSH.

### 3.9. Particle Storage Stability

**Figure 7:**
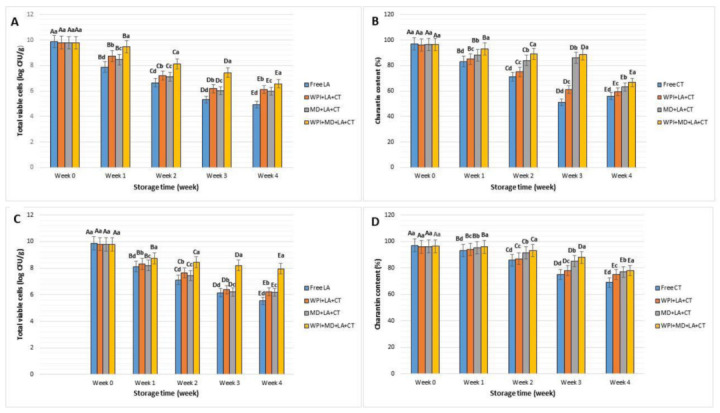
Storage stability at 25 °C for LA (**A**), CT (**B**), and at 4 °C for LA (**C**), CT (**D**). WPI + LA + CT (**A**), MD + LA + CT (**B**), WPI + MD + LA + CT (**C**). WPI + LA + CT: Whey protein isolate + *L. acidophilus +* Charantin*;* MD + LA + CT: Maltodextrin + *L. acidophilus* + Charantin; WPI + MD + LA + CT: Whey protein isolate + *L. acidophilus* + Maltodextrin + *L. acidophilus +* Charantin. Data represent means ± standard deviation. Different uppercase letters above each column represent significant differences for a sample between storage weeks (*p* < 0.5), and lowercase letters above each column indicate significant differences between samples within a week. (*p* < 0.5).

There is an increasing demand for food products with enhanced functional properties. However, maintaining the integrity of probiotic cells and bioactive compounds upon storage remains a challenge. Hence, the effects of encapsulation on LA viability and CT content during storage at room (25 °C) and refrigerated (4 °C) temperatures were studied, and results are reported in Figure 7. From the first week and throughout the storage period at 25 °C, probiotic cells uniformly experienced a loss of viability (Figure 7A), which was attributed to the effect of oxidation of membrane lipids and denaturation of proteins causing degradation of macromolecules in bacterial cells [78]. Nonetheless, encapsulated bacteria exhibited a moderate loss compared to free cells which accounted for half of their initial count at the end of the 30 days of storage at 25 °C. It was hypothesized that the protective effect of wall materials and the antioxidative properties of CT may have led to this outcome. Moreover, among the encapsulated samples, WPI + MD + LA + CT had the highest count of viable cells, indicating the effectiveness of the blended material to protect the probiotic cellular structure against oxidation occurring during storage at room temperature. Besides, the CT content when stored at 25 °C (Figure 7B) remained stable till the end of the storage period in encapsulated samples compared to free samples which presented a continuous decrease throughout the storage period due to oxidation. In addition, WPI + MD + LA + CT exhibited the highest retention of CT content at 25 °C. In this case, the formation of H-bonding between the carrier materials may have increased the airproof property of WPI + MD + LA + CT microparticles [79]. During storage at 4 °C, encapsulated cells were still in sufficient number after the 30 days of storage compared to free cells that lost their viability after 15 days (Figure 7C). Similarly, CT content was higher in microparticles than in its free form (Figure 7D). Microparticles produced with blended carrier materials WPI + MD showed a higher viable count for LA (7.95 ± 0.1 log CFU/g) and higher content of CT (78%) at the end of the storage period. Hence, the aforementioned findings suggest that microsphere WPI + MD would be an effective carrier material to maintain the integrity of probiotic-bioactive compounds and synbiotic systems upon storage. Finally, it is to be noted that further investigations under diverse storage conditions (low temperature, lyophilization, etc.) would provide more information about the storage stability of synbiotic microparticles.

## 4. Conclusions

In this study, probiotic *L. acidophilus* and Charantin were co-encapsulated for the first time using WPI, MD, and their combination WPI + MD, in 1:1 core ratio, via freeze-drying technology. Blended carrier microparticles WPI + MD presented higher encapsulation efficiency than single-carrier encapsulates WPI and MD. The physicochemical characteristics of microparticles were found acceptable according to established standards for powder stability. Besides, WPI + MD provided superior protection to LA and CT upon in vitro digestion, while the surface hydrophobicity of cells was significantly enhanced. Additionally, FTIR analysis revealed the formation of strong hydrophobic bonds between carrier materials, and TGA-DSC analysis unveiled the higher thermal stability of microparticles WPI + MD compared to single carrier materials. Moreover, microparticles WPI + MD exhibited higher in vitro antioxidant activity, α-amylase inhibitory activity, and lipase inhibitory activity. Furthermore, WPI + MD remarkably improved LA viability and CT content retention upon 30 days of storage at 4 °C. Hence, synbiotic microparticles WPI + MD + LA + CT are a very promising strategy to optimize probiotic and bioactive compounds functionality and food application.

## Figures and Tables

**Table 1:** Detailed composition of stock solutions used to prepare simulated digestive fluids.

Components	Stock Concentration (M)	SSF (pH 7.0)	SGF (pH 3.0)	SIF (pH 7.0)
		Concentration (mM)	Concentration (mM)	Concentration (mM)
KCl	0.5	20.1	6.9	6.8
KH_2_PO_4_	0.5	8.7	0.9	0.8
NaHCO_3_	1	23.6	25	91
NaCl	2	-	47.2	38.4
MgCl_2_(H_2_O)_6_	0.15	0.3	0.12	0.33
(NH_4_)_2_CO_3_	0.5	0.12	0.5	-

The final volume was adjusted to 500 mL with distilled water for each simulated fluid. SSF = Simulated Salivary Fluid, SGF = Simulated Gastric Fluid, SIF = Simulated Intestinal Fluid.

**Table 2:** Physicochemical characteristics of microparticles WPI + LA + CT, MD + LA + CT, and WPI + MD + LA + CT. Whey protein isolate + *L. acidophilus* + Charantin; MD + LA + CT: Maltodextrin + *L. acidophilus* + Charantin; WPI + MD + LA + CT: Whey protein isolate + Maltodextrin+ *L. acidophilus* + Charantin.

	Moisture Content (%)	Water Activity	Hygroscopicity (%)	Particles Mean d (4, 3) μm	ζ-Potential
WPI + LA + CT	4.05 ± 0.3 ^c^	0.59 ± 0.5 ^c^	14.25 ± 0.4 ^c^	52.637 ± 5.42 ^b^	2.65 ± 0.5
MD + LA + CT	3.25 ± 0.4 ^a^	0.22 ± 0.1 ^a^	11.92 ± 0.3 ^a^	50.393 ± 1.26 ^a^	−2.31 ± 0.3
WPI + MD + LA + CT	3.39 ± 0.2 ^b^	0.41 ± 0.2 ^b^	12.15 ± 0.1 ^b^	68.412 ± 3.22 ^c^	4.92 ± 0.2

All values are mean ± standard deviation. Superscript letters (^a–c^) in the same column with different letters indicate significant differences (*p* < 0.05).
